# Putative RNA Ligase RtcB Affects the Switch between T6SS and T3SS in *Pseudomonas aeruginosa*

**DOI:** 10.3390/ijms222212561

**Published:** 2021-11-22

**Authors:** Maryam Dadashi, Lin Chen, Ahmad Nasimian, Saeid Ghavami, Kangmin Duan

**Affiliations:** 1 Department of Oral Biology, Rady Faculty of Health Sciences, Dr. Gerald Niznick College of Dentistry, University of Manitoba, Winnipeg, MB R3E 0W2, Canada; Dadashim@myumanitoba.ca; 2 College of Life Sciences, Northwest University, Xi’an 710069, China; Chenlin@nwu.edu.cn; 3 Department of Human Anatomy and Cell Science, Rady Faculty of Health Sciences, Max Rady College of Medicine, University of Manitoba, Winnipeg, MB R3E 0W2, Canada; Nnasimian@gmail.com (A.N.); Saeid.Ghavami@umanitoba.ca (S.G.); 4 Department of Medical Microbiology and Infectious Disease, Rady Faculty of Health Sciences, University of Manitoba, Winnipeg, MB R3E 0W2, Canada

**Keywords:** virulence factors, T6SS, T3SS, c-di-GMP, *P. aeruginosa*, microbial competition, plant infection

## Abstract

The opportunistic pathogen *Pseudomonas aeruginosa* is a significant cause of infection in immunocompromised individuals, cystic fibrosis patients, and burn victims. To benefit its survival, the bacterium adapt to either a motile or sessile lifestyle when infecting the host. The motile bacterium has an often activated type III secretion system (T3SS), which is virulent to the host, whereas the sessile bacterium harbors an active T6SS and lives in biofilms. Regulatory pathways involving Gac-Rsm or secondary messengers such as c-di-GMP determine which lifestyle is favorable for *P. aeruginosa*. Here, we introduce the RNA binding protein RtcB as a modulator of the switch between motile and sessile bacterial lifestyles. Using the wild-type *P. aeruginosa* PAO1, and a *retS* mutant PAO1(∆*retS*) in which T3SS is repressed and T6SS active, we show that deleting *rtcB* led to simultaneous expression of T3SS and T6SS in both PAO1(∆*rtcB*) and PAO1(∆*rtcB*∆*retS*). The deletion of *rtcB* also increased biofilm formation in PAO1(∆*rtcB*) and restored the motility of PAO1(∆*rtcB*∆*retS*). RNA-sequencing data suggested RtcB as a global modulator affecting multiple virulence factors, including bacterial secretion systems. Competitive killing and infection assays showed that the three T6SS systems (H1, H2, and H3) in PAO1(∆*rtcB*) were activated into a functional syringe, and could compete with *Escherichia coli* and effectively infect lettuce. Western blotting and RT-PCR results showed that RtcB probably exerted its function through RsmA in PAO1(∆*rtcB*∆*retS*). Quantification of c-di-GMP showed an elevated intracellular levels in PAO1(∆*rtcB*), which likely drove the switch between T6SS and T3SS, and contributed to the altered phenotypes and characteristics observed. Our data demonstrate a pivotal role of RtcB in the virulence of *P. aeruginosa* by controlling multiple virulence determinants, such as biofilm formation, motility, pyocyanin production, T3SS, and T6SS secretion systems towards eukaryotic and prokaryotic cells. These findings suggest RtcB as a potential target for controlling *P. aeruginosa* colonization, establishment, and pathogenicity.

## 1. Introduction

*P. aeruginosa* is a motile Gram-negative bacterium and a nosocomial pathogen causing life-threatening infections in humans [1]. Its large genome and numerous regulatory elements allow the bacterium to thrive in versatile environments [2].

*P. aeruginosa* produces a variety of virulence factors, including cell-associated structural features such as flagellum and pili, and extracellular effectors such as exotoxins and pyocyanin [3]. These exotoxins are secreted to the environment or directly to the host and/or other bacteria [4,5,6]. During the early stages of infection, the bacterial pathogen in a planktonic and motile lifestyle induces tissue damage and inflammation in the host [7]. The bacterium can physically connect with the host and initiate the translocation of toxins directly to the host tissue through the type three secretion system (T3SS) [8]. Together with LasB protease, T3SS effectors damage host cell integrity, and lead to rapid bacterial growth and spread, resulting in an acute infection of the host [7]. Under conditions of chronic infection, the bacterium forms a mucoid matrix and loses its motility and T3SS activity, rendering a sessile biofilm-forming stage [9]. Biofilm formation promotes long-term bacterial survival and the development of resistance to clearance by the host immune system and drug interventions [7].

The phosphorylation of GacA leads to an upregulation of two small RNAs (sRNAs), RsmY and RsmZ. RsmY/Z sRNAs bind to the global post-transcriptional regulator RsmA and sequester this protein, resulting in the expression of type six secretion systems (T6SSs) and biofilm formation-related genes. The sensor kinase RetS modulates the activity of GacS, which, in turn, inhibits the phosphorylation of GacA and represses the transcription of RsmY/Z. Without antagonizing sRNAs, RsmA promotes the expression of T3SS genes and negatively affects T6SS [10,11,12,13,14]. The sessile lifestyle of *P. aeruginosa*, marked by biofilm production and higher pyocyanin pigmentation, was reported as a specific characteristic of PAO1(∆*retS*) [10]. Signaling associated with cyclic diguanylate (c-di-GMP) has emerged as a key mediator of the switch between the motile and sessile *P. aeruginosa* lifestyles by controlling features such as flagella rotation, antibiotic resistance, adhesin expression, type four pili retraction, secondary messengers, and stress responses [15,16]. C-di-GMP biogenesis is responsive to cellular stress and can promote or prevent either lifestyle of the bacteria [17].

The stress response protein RtcB is a conserved protein in all three domains of life [18]. In bacteria, it is believed to splice the 5′-OH ends of RNA to either 2′, 3′-cyclic phosphate or 3′-phosphate ends [19]. In *E. coli*, RtcB re-ligates a fragment of 16S rRNA to the rRNA body after cleavage by a stress-induced endonuclease [20]. In *P. aeruginosa*, RtcB is encoded by an operon containing an RNA cyclase RtcA and two conserved proteins of unknown function; PA4582 and PA4584 [21,22]. The operon transcription is regulated by the adjacent protein RtcR, encoded in the opposite direction [21]. The activation of RtcR and its divergent CARF (CRISPR-associated Rossman fold) domain were recently discussed [23,24]. CARF is a ligand-binding regulator [25], and damaged tRNAs activate the transcription of the *rtcBA* operon in the presence of RtcA [26]. It was suggested that the highly conserved RtcB might play a vital role in bacterial survival [18]. There is no report so far on the role of RtcB in the virulence of bacteria.

In this study, we identified RtcB as a modulator of T6SSs and T3SS expression. We explored the role of RtcB beyond its stress response activity (RNA 2′, 3′-cyclic phosphate, and 5′-OH ligase) in *P. aeruginosa*. We also investigated the role of RtcB *P. aeruginosa* in pathogenicity and the underlying mechanisms using the wild type *P. aeruginosa* PAO1, which has an active T3SS and inactive T6SS under normal lab conditions, and a *retS* mutant PAO1(∆*retS*), in which T3SS is repressed and T6SS is constitutively active. RNA-sequencing was also performed to examine the global effect of RtcB.

## 2. Results

### 2.1. RtcB Controls the Expression of Multiple Phenotypes and Virulence Factors

The conserved RtcB protein is involved in the repair of stress-induced RNA damage [18,27,28]. Protein blast analysis of RtcB in *E. coli* and *P. aeruginosa* showed that the proteins are 70% identical, thus expectedly fulfilling similar functions [18]. Since the role of RtcB in the virulence of bacteria has not yet been fully explored, we examined the possible function of RtcB in the pathogenicity of *P. aeruginosa*. The PAO1(∆*rtcB*) in wild type background PAO1, and PAO1(∆*rtcB*∆*retS*) in PAO1(Δ*retS*) background, were generated to investigate the role of RtcB in both motile and sessile bacteria, respectively. In PAO1(Δ*retS*), multiple virulence factors are down-regulated, including T3SS [10,29,30,31].

**Figure 1:**
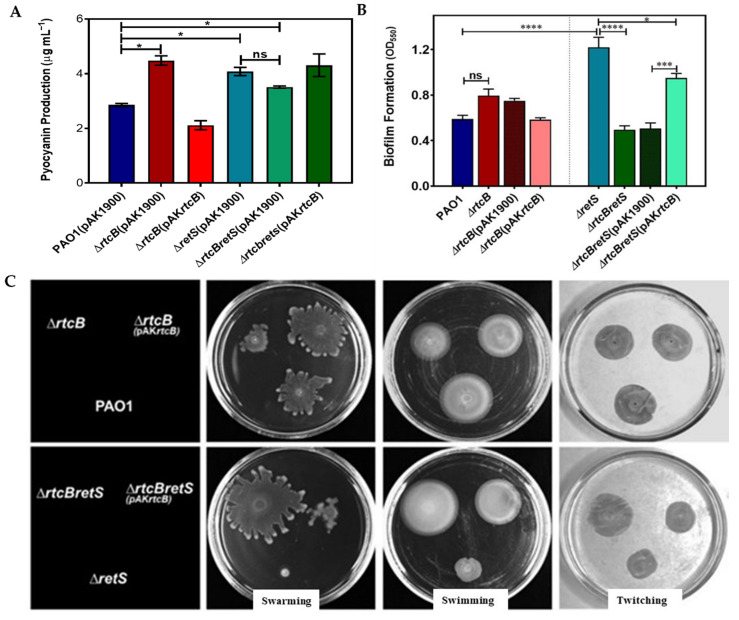
Deletion of *rtcB* influenced multiple virulence factors of *P. aeruginosa*. (**A**) Measurement of pyocyanin production in PAO1, PAO1(∆*rtcB*), PAO1(∆*retS*), and PAO1(∆*rtcB*∆*retS*), and the complemented deletion strains with *rtcB* expression vector. Pyocyanin was produced in significantly higher amounts in PAO1(∆*rtc*B) and PAO1 (∆*retS*) compared to PAO1. The complementation of *rtcB* deletion mutants restored the pyocyanin production. (**B**) Biofilm formation was assayed in a 96-multi-well plate under static conditions. Biofilm formation was significantly higher in PAO1(∆*retS*) than in PAO1. The PAO1(∆*rtcB*∆*retS*) strain formed significantly less biofilms compared to PAO1(∆*retS*). Biofilm formation in the complemented strains returned to the levels of PAO1 and PAO1(∆*retS*), respectively. (**C**) Motility assays revealed that swarming and swimming motilities of PAO1(∆*rtcB*) were decreased compared to the wild-type PAO1. Swarming, swimming, and twitching were increased in PAO1(∆*rtcB*∆*retS*) compared with PAO1(∆*retS*). Overexpression of *rtcB* in the *rtcB* deletion strains reduced the motilities to levels similar to the background. (**A**,**B**): Data from three independent experiments were analyzed using one-way ANOVA with Dunnette post-hoc test. (**C**): images are representative of three independent experiments. ns, *p* > 0.05, * *p* < 0.05, *** *p* < 0.001, and **** *p <* 0.0001.

We tested multiple phenotypes of PAO1(∆*rtcB*) and PAO1(∆*rtcB*∆*retS*), including pyocyanin production, biofilm formation, and motility, as compared with PAO1 and PAO1(∆*retS*), respectively. The first noticeable difference was that PAO1(∆*rtcB*) cultures showed a significantly higher production of pyocyanin compared to PAO1 [32]. To evaluate the effects of *rtcB* deletion on pyocyanin production in these cells, the double mutant PAO1(∆*rtcBretS*) was constructed. Subsequently, pyocyanin biosynthesis was assayed in the PAO1, PAO1(∆*rtcB*), PAO1(∆*retS*), and PAO1(∆*rtcBretS*) strains. Although production of pyocyanin increased in PAO1(∆*rtcB*) compared to PAO1, there was no significant difference (*p* = 0.433) between PAO1(∆*rtcB*∆*retS*) and PAO1(∆*retS*) (Figure 1A). We further tested the impact of *rtcB* deletion on the biofilm production and motility of *P. aeruginosa.*

Interestingly, biofilm formation in PAO1(∆*rtcB*∆*retS*) was reduced to wild-type levels, indicating that the increase due to deletion of *retS* was normalized by *rtcB* deletion (*p <* 0.0001) (Figure 1B). It appeared that PAO1(∆*rtcB*) formed slightly more biofilm; however, this did not reach statistical significance.

Bacterial motility is the hallmark of the acute phase of infection and is importantly controlled by the complex regulatory interplay of flagellar assembly, chemotaxis, type four pili, the Gac-Rsm two-component system, and quorum sensing [33,34,35,36,37]. As shown in Figure 1C, twitching motility did not show an apparent change in PAO1(∆*rtcB*), whereas it increased in PAO1(∆*rtcB*∆*retS*) compared to PAO1(∆*retS*). Swarming and swimming of PAO1(∆*rtcB*) were decreased compared to wild type PAO1 and were highly elevated in PAO1(∆*rtcBretS*) compared to background PAO1(∆*retS*).

### 2.2. Putative RNA Ligase RtcB Affects T6SS and T3SS Transcription and Its Deletion Inactivates the Gac-Rsm Controlled Switch between the Two Secretion Systems

**Figure 2:**
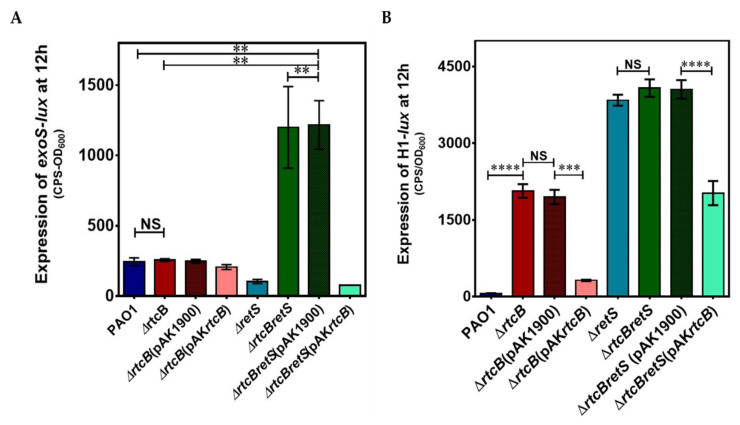
The promoter activities of *exoS* (**A**), an effector of T3SS, and H1-T6SS (**B**) in PAO1 and its derivative mutants at the 12 h growth time point. CTX-H1 and CTX-*exoS* reporter fusions were integrated into the chromosome of *P. aeruginosa.* Complementing vector pAK1900-*rtcB* was introduced to the mutant strains PAO1(∆*rtcB*) and PAO1(∆*rtcB*∆*retS*), respectively, to construct the complementation strains. Expression is reported as light production (counts per second) normalized to growth (OD_600_). The results shown are the average of three independent readouts performed in triplicate. Data were analyzed using one-way ANOVA with Dunnette post-hoc test. NS *p* > 0.05, ** *p* < 0.01, *** *p* < 0.001, and **** *p* < 0.0001.

Lower biofilm formation and higher motility concomitant with T3SS expression have been repeatedly reported as typical characteristics of motile bacteria [37,38]. Moreover, when T3SS is activated under the Gac-Rsm system, T6SS is repressed in an opposite manner. To assess if lower biofilm formation in PAO1(∆*rtcBretS*) is the result of a switch to a motile lifestyle due to active T3SS, the transcriptional activity of T3SS was examined in the PAO1, PAO1(∆*rtcB*), PAO1(∆*retS*), and PAO1(∆*rtcBretS*) strains. No differences were observed in the expression of *exoS*, an effector of T3SS, between PAO1 (∆*rtcB*) and the wild-type strain. Interestingly, while deletion of *retS* alone resulted in a moderate decrease in *exoS* expression, combined deletion of *rtcB* and *retS* dramatically increased its abundance (Figure 2A). We also measured the transcription of the T6SS reporter, using H1-T6SS as a representative of T6SSs, to verify if T6SS is repressed in PAO1(∆*rtcBretS*). While no change was observed in H1-T6SS promoter activity in PAO1(∆*rtcBretS*), a considerable increase could be detected in PAO1(∆*rtcB*) (*p* < 0.0001). The active T3SS and T6SS in *rtcB* deletion mutants suggested that the switch between the activation of T3SS and T6SS by Gac-Rsm pathway was voided by the deletion of *rtcB,* and both secretion systems are expressed in PAO1(∆*rtcB*) and PAO1(∆*rtcB*∆*retS*) simultaneously (Figure 2B). These results show a pivotal role of RtcB in the control of some of the major virulence factors of *P. aeruginosa*.

### 2.3. Transcriptional Profiling of the rtcB Knockout Mutant

To further investigate the impact of RtcB on the expression dynamics associated with T3SS and T6SS activities in *P. aeruginosa*, RNAseq analysis was carried out in two groups: (1) PAO1 vs. PAO1(∆*rtcB*) and (2) PAO1(∆*retS*) vs. PAO1(∆*rtcB*∆*retS*).

**Figure 3:**
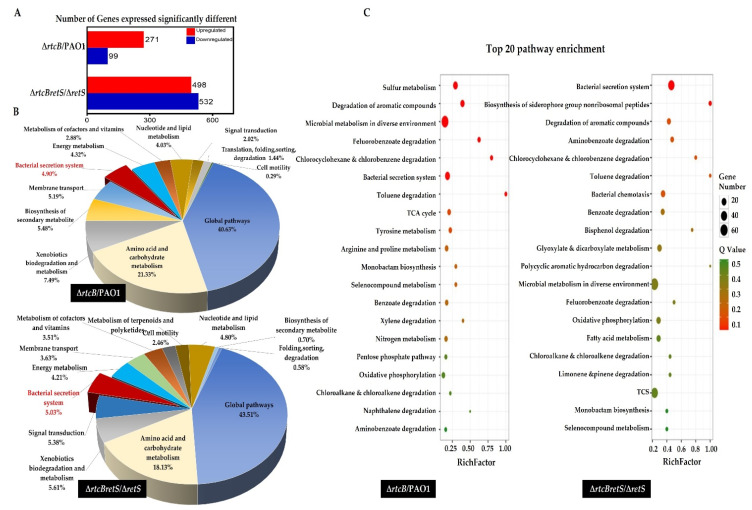
The effect of rtcB deletion on the transcriptome of P. aeruginosa. (**A**) A total of 370 genes showed significantly altered expression when comparing PAO1(∆*rtcB*) to PAO1 (group 1), while 1030 genes were differentially expressed between PAO1(∆*rtcB*∆*retS*) and PAO1(∆*retS*) (group 2). (**B**) The pie charts represent the Gene Ontology analysis of DEGs in groups 1 (upper panel) and 2 (lower panel). In group 1, 80.68% of DEGs belonged to global and metabolic pathways. The largest group of genes after metabolic-related genes were membrane transport genes (5.19%) and bacterial secretion systems (shown as a separate slice) (4.90%). In group 2, a similar pattern was observed, with the biggest difference belonging to the signal transduction (5.38%) and bacterial secretion systems (5.03%). (**C**) KEGG enrichment in groups 1 (**left** panel) and 2 (**right** panel). The Y-axis shows the top 20 pathways affected by the deletion of *rtcB*. The X-axis represents the ratio of the number of enriched DEGs in the categorized KEGG to the total genes of that category. Dot size represents the number of DEGs in the pathway, while dot color indicates the Q-value. The Q-value is the enrichment level of each category and represents how significant genes are affected in *rtcB* deficient strains. In group 1, the only virulence-related pathway shown in the KEGG plot was bacterial secretion systems; in group 2, the expression of genes associated with bacterial secretion systems and bacterial chemotaxis was significantly different.

Differentially expressed genes (DEGs) across samples with fold change ≥±2 (log 2 fold change ≥±1) and false discovery rate-adjusted *p* (*q* value) < 0.05 were identified by edgeR package (http://www.r-project.org/; accessed on 11 January 2019). The effect of *rtcB* deletion on gene expression of the whole *P. aeruginosa* genome is presented in Supplementary Materials, Tables S1 and S2. DEGs of the transcriptome analysis showed that a total of 370 genes were differentially expressed in group 1: 271 genes were upregulated, and 99 genes were downregulated (Supplementary Materials, Table S3). In group 2, 1030 DEGs were revealed, of which 498 were up- and 532 were downregulated (Figure 3A) (Supplementary Materials, Table S4). The DEGs filtered by significance of log 2-fold change are shown in Supplementary Materials, Tables S5 and S6.

DEGs were specified based on the sequence homology and the gene ontology (GO) of those that were significantly enriched (*p* < 0.05). Comparing results to the Kyoto encyclopedia of genes and genomes (KEGG), enrichments indicated that the transcription of genes participating in cellular metabolic processes, including global pathways, the metabolism of carbohydrates and amino acids, and biodegradation of xenobiotics, were significantly altered in both groups. This confirms that RtcB plays a key role in the modulation of the cell metabolism. Also, KEGG enrichments show that deletion of *rtcB* affects secondary metabolites, signal transduction pathways, and cell motility. Of particular interest to our study, bacterial secretion systems (a subcategory of transmembrane proteins) represent the sixth and fifth most affected DEGs in groups 1 and 2, respectively. That includes T2SS, T5SS, T3SS, T6SS, and Sec-SRP in both groups. The top 20 KEGG enrichments modulated by the deletion of *rtcB* are presented in Figure 3C for groups 1 (left panel) and 2 (right panel). DEGs related to bacterial secretion systems T2SS, T5SS, and Sec-SRP are outlined in Table 1, whereas those associated with T3SS and T6SS are shown separately.

### 2.4. Deletion of rtcB Does Not Affect Transcription of rsmA but Upregulates the Expression of clpV1, hcp1, exsC, and exsA

To confirm the RNA-sequencing data, we selected various genes of T3SS and T6SS, and quantified those by RT-PCR: *hcp1*, *clpV1,* and *rsmA* for PAO1(∆*rtcB*) vs. PAO1, and *exsA*, *exsC*, and *rsmA* for PAO1(∆*rtcB*∆*retS*) vs. PAO1(∆*retS*).

**Figure 4:**
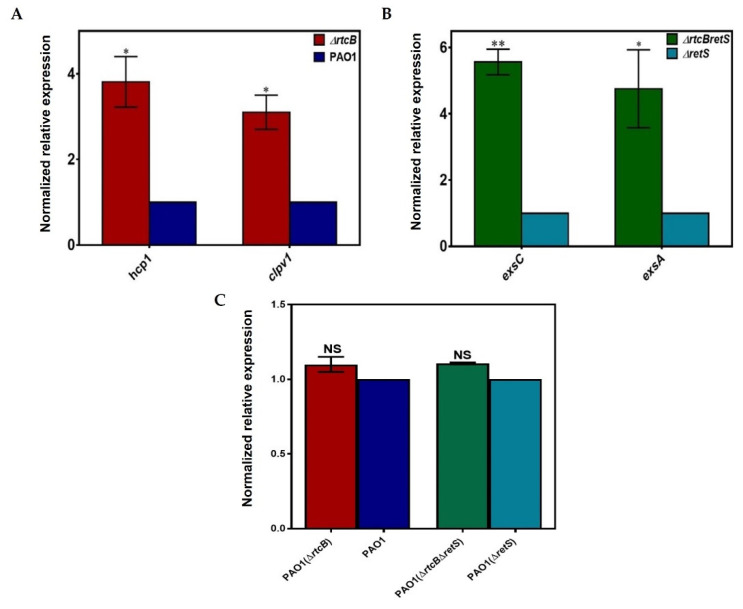
Confirmation of RtcB effect on rsmA and genes associated with H1-T6SS and T3SS. The mRNA levels of H1-T6SS and T3SS genes were quantified by qPCR. (**A**) The transcription levels of clpV1 and *hcp1* were significantly higher in PAO1(∆*rtcB*) than in PAO1 (* *p* < 0.05). (**B**) The expression of *exsA* and *exsC* was upregulated considerably in PAO1(∆*rtcB*∆*retS*) compared to PAO1(∆*retS*) (* *p* < 0.05 and ** *p* < 0.01). (**C**) The expression level of *rsmA* remained unchanged in all strains, confirming the RNAseq data. Expression levels were normalized to the levels of housekeeping gene *rpoD* and presented as a fold change relative to the control (=1). Data were analyzed using unpaired Student’s *t*-test. Two independent experiments were performed in triplicate. NS *p* > 0.05.

Hcp1 secretion is an indication of active H1-T6SS [39] and ClpV1 is the ATPase of H1-T6SS required for the phosphorylation-dependent secretion of H1-T6SS [40]. Gene expression relative to the *rpoD* housekeeping gene was analyzed. The results showed that the mRNA levels of *clpV1* and *hcp1* were increased more than 3- and 3.5-fold, respectively, in PAO1(∆*rtcB*) compared to PAO1 (* *p* < 0.05, Figure 4A). In PAO1(∆*rtcB*∆*retS*), the expression of *exsA* and *exsC* was 4.5- (* *p* < 0.05) and 5.5-fold (** *p* < 0.01) higher than in PAO1(∆*retS*) (Figure 4B). The transcription of *rsmA* remained unchanged upon the deletion of *rtcB* in both PAO1 and PAO1(∆*retS*) (Figure 4C).

### 2.5. The Three T6SSs Are Activated upon Deletion of rtcB and Promote the Pathogenicity of P. aeruginosa

**Figure 5:**
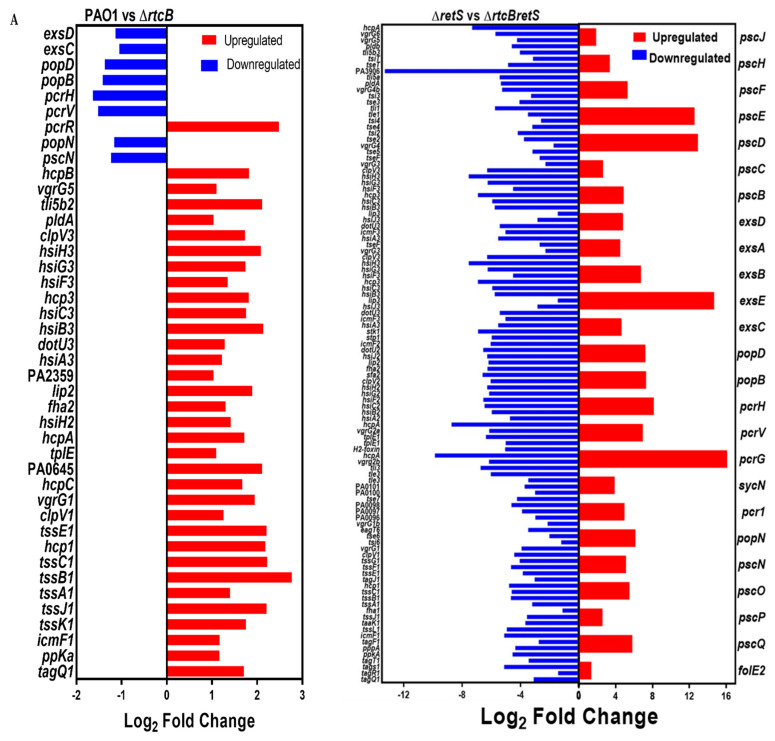

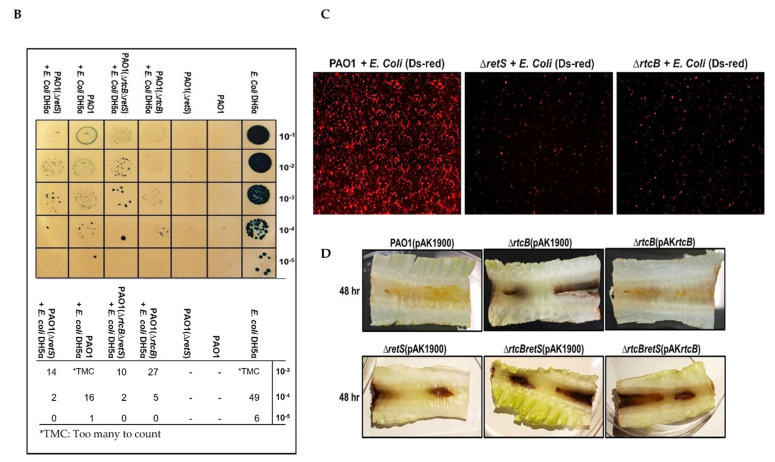
Functional T6SS in ∆*rtcB* participated in the killing of *E. coli* and infection of lettuce leaves. (**A**) Differential expression of T3SS and T6SS genes in PAO1 vs. PAO1(∆*rtcB*) and PAO1(∆*retS*) vs. PAO1(∆*rtcB*∆*retS*). (**B**) Competitive prokaryotic killing assay, in which co-cultures of *E. coli* with predator *Pseudomonas* strains were spotted on coliform agar plates. The blue color represents live *E. coli* cells. The table shows the colony forming unit (CFU) at 10^−5^, 10^−4^, and 10^−3^ dilutions. Lower CFU of live *E. coli* cells (blue colonies) in co-cultures of PAO1(∆*rtcB*)+*E. coli*, in comparison with co-cultures of PAO1+*E. coli* strains, represent the higher killing potential of PAO1(∆*rtcB*) with activated T6SSs. (**C**) Fluorescence competition assay showing live *E. coli* harboring *pilG*-DsRed fluorescent reporter in red. (**D**) Photographed lettuce midribs show the infection sites after 48 h. PAO1(∆*rtcB*) and PAO1(∆*rtcB*∆*retS*) caused a more robust infection than PAO1 and PAO1(∆*retS*), respectively.

Transcriptomics showed that all three T6SSs (H1-, H2-, H3-T6SS) are affected by *rtcB* deletion. Figure 5A represents the expression of specific T6SSs and T3SS genes significantly affected by deletion of *rtcB*. To confirm the sequencing data and assess the involvement of RtcB in the pathogenicity of *P. aeruginosa*, we performed a competition assay for both prokaryotic [41,42] and eukaryotic prey [43,44] targeted by H1-, H2-, and H3-T6SS, respectively. In the prokaryotic competition assay, live *E. coli* prey cells not killed by *P. aeruginosa* appear blue in the colonies. Thus, a higher blue color intensity indicates an increased survival rate of *E. coli* due to less virulence of *P. aeruginosa*. PAO1(∆*retS*) harbors constitutively active T6SS (T6SS^+^) and was used as a positive control. As expected, PAO1(∆*retS*) exhibited significantly higher virulence compared to wild-type PAO1, as evidenced by a lower survival rate of *E. coli* cells. Importantly, PAO1(∆*rtcB*) also showed a higher killing potential than PAO1, similar to that of PAO1(∆*retS*), implying that T6SS in PAO1(∆*rtcB*) is activated (Figure 5B). To verify the increased pathogenicity of PAO1(∆*rtcB*) toward prokaryotes, we conducted a fluorometric killing assay in which prey *E. coli* cells were transformed with a vector harboring a *pilG*-DsRed fluorescent reporter; the density of red fluorescence is representative of viable *E. coli*. Fully confirming the results of our initial killing assay, PAO1(∆*rtcB*) demonstrated a similar killing force to PAO1(∆*retS*). These findings imply that T6SS is functionally active in PAO1(∆*rtcB*) and able to kill *E. coli* cells at a higher rate than PAO1 (Figure 5C).

Next, we performed a eukaryotic (plant) infection assay to validate the functionality of H2-T6SS and H3-T6SS by deletion of *rtcB*. Lettuce midribs inoculated with PAO1(∆*rtcB*) were highly infected, as shown by a much darker infection site color as compared to leaves exposed to PAO1. Furthermore, PAO1(∆*rtcB*∆*retS*) appeared to exhibit a slightly higher virulence toward the plant in comparison with PAO1(∆*retS*) (Figure 5D). These results further support that T6SS is activated after the deletion of *rtcB* in PAO1.

### 2.6. RsmA Protein Concentration Is Increased by Deletion of rtcB

**Figure 6:**
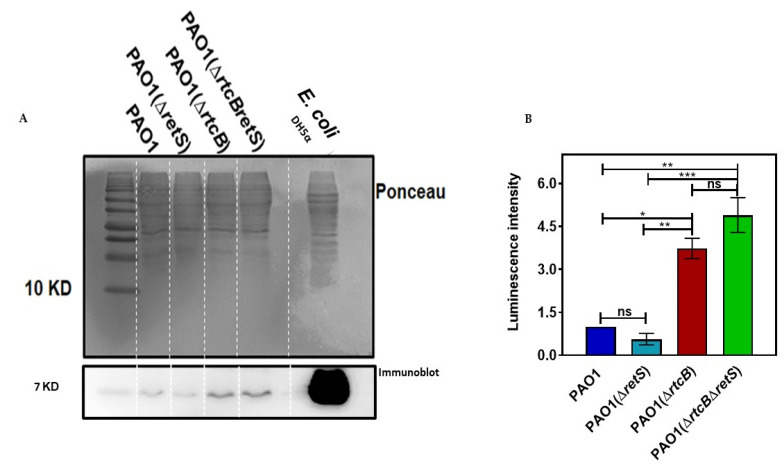
RsmA protein abundance was increased in PAO1(∆rtcB) and PAO1(∆rtcB∆retS), compared to their respective controls. (**A**) The total protein content of each strain was determined by Bradford assay, separated by SDS-PAGE, and probed with the E. coli CsrA antibody. Ponceau staining was used as a loading control. The RtcB deficient strain showed an increased content of RsmA compared with PAO1 and PAO1(∆*retS*). (**B**) Densitometric analysis of the protein bands showed that the amount of RsmA in PAO1(∆*rtcB*) and PAO1(∆*rtcB*∆*retS*) was significantly increased. The luminescence intensity was measured with ImageJ software. Results are the average of three independent experiments. Data were analyzed using one-way ANOVA with Dunnette post-hoc test. ns *p* > 0.05, * *p* < 0.05, ** *p* < 0.01, and *** *p* < 0.001.

To explore how *rtcB* deletion modulates the transcription of T3SS and T6SS genes, we performed immune blotting experiments to determine the expression of the RNA binding protein RsmA. As indicated earlier, mRNA expression of RsmA was unaltered by *rtcB* deletion in both strains; however, RsmA function is post-translationaly regulated by RsmY, RsmZ, and RsmV sRNAs [45,46]. Western blot analysis revealed that protein expression of RsmA was markedly increased in PAO1(∆*rtcB*) and PAO1(∆*rtcB*∆*retS*), as compared to their respective controls (Figure 6), while the transcription of *rsmA* was unaffected by the deletion of *rtcB*. These data could explain the increase in *exoS* reporter activity in PAO1(∆*rtcB*∆*retS*) (Figure 2A,B), as more RsmA would increasingly activate T3SS. However, the expected outcome of increased RsmA in PAO1(∆*rtcB*) as a repressor of T6SSs would be the downregulation of H1-T6SS. These results suggest that RtcB exerts its function on secretion systems through multiple pathways.

### 2.7. Complementation of PAO1(∆rtcB∆retS) with pAKrsmY and pAKrsmY Diminishes the Expression of T6SS and Represses T3SS

**Figure 7:**
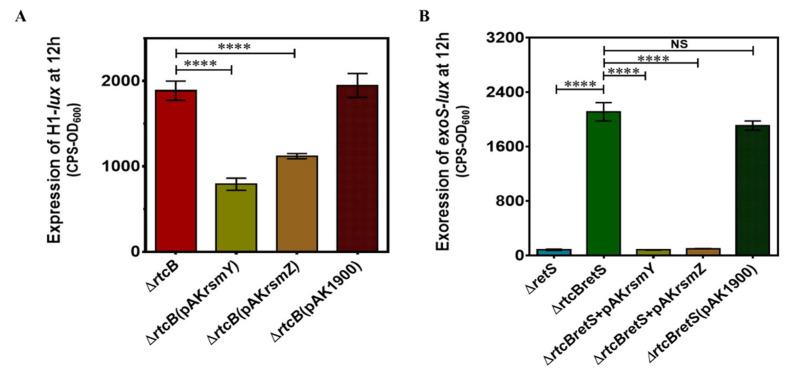
Complementation of *rtcB* deletion mutants with RsmY/Z overexpressing vectors altered H1-T6SS and *exoS* reporter activity. Expression of H1-T6SS (**A**) and *exoS* (**B**) was measured in mutant strains. Data were analyzed using one-way ANOVA with Dunnette post-hoc test, NS *p* > 0.05, **** *p* < 0.0001.

We generated strains of PAO1(∆*rtcB*), PAO1(∆*rtcB*∆*retS*), and PAO1(∆*retS*) overexpressing *rsmY* and *rsmZ* sRNAs to assess the reporter activity of H1-T6SS and *exoS* as a representative of T6SSs and T3SS, respectively. The overexpression of RsmY/Z lowered the reporter activity of H1-T6SS in PAO1(∆*rtcB*). The result confirmed that the upregulation of H1-T6SS in PAO1(∆*rtcB*) is probably as a result of more RsmY/Z sequestering the RsmA protein, instead of a change in RsmA protein amount (Figure 7A).

However, the complementation of PAO1(∆*rtcB*∆*retS*) with RsmY/Z overexpression vectors repressed the expression of T3SS (Figure 7B). The upregulation of T3SS in PAO1(∆*rtcB*∆*retS*) is achieved through the downregulation of RsmY/Z by the deletion of *rtcB*, resulting in increased RsmA and activation of T3SS. The results suggest that the RtcB protein might modulate H1-T6SS and T3SS activity through different pathways. This is perhaps the reason that both H1-T6SS and T3SS secretion systems remain active in PAO1(∆*rtcB*) and PAO1(∆*rtcB*∆*retS*).

### 2.8. Intracellular Levels of c-di-GMP Are Significantly Increased in rtcB Deletion Mutant

**Figure 8:**
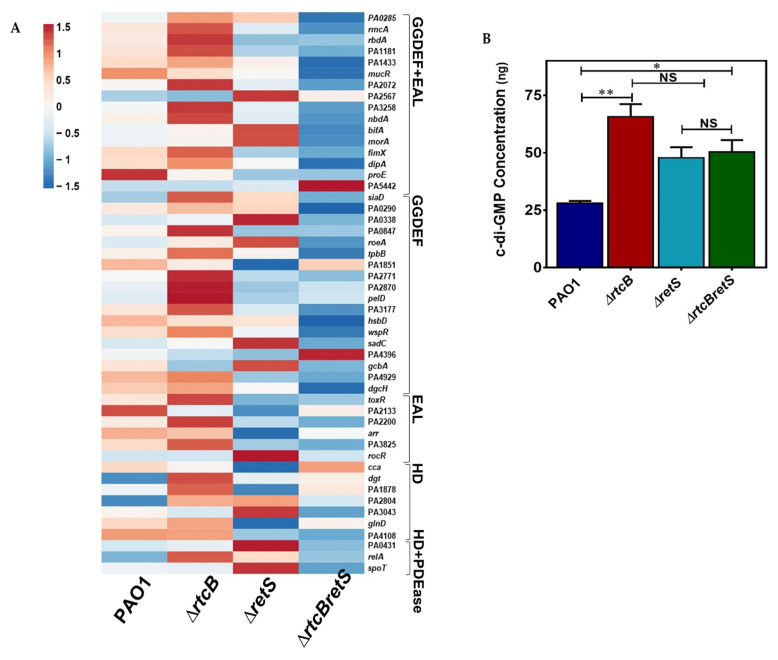
Comparison of c-di-GMP-related transcriptome and intracellular c-di-GMP concentration in PAO1, PAO1(∆*rtcB*), PAO1(∆*retS*), and PAO1(∆*rtcB*∆*retS*) strains. (**A**) The transcription levels of genes involved in c-di-GMP secondary messenger metabolism were calculated from FPKM values using the MATLAB graphical toolbox and represented as color-coded boxes (blue, downregulation; red-code, upregulation). (**B**) Deletion of *rtcB* significantly increased the intracellular concentration of c-di-GMP (PAO1 vs. PAO1(∆*rtcB*)), indicating an important role of *rtcB* in the modulation of c-di-GMP metabolism. Data were analyzed using one-way ANOVA with Dunnette post-hoc test, NS *p* > 0.05, * *p* < 0.05, ** *p* < 0.01.

Our RNA sequencing data showed that several genes associated with the synthesis and degradation of c-di-GMP are differentially expressed upon deletion of *rtcB*. Those genes are represented in a heatmap graph plotted by MATLAB graphical toolbox (Figure 8A). A higher rate of c-Di-GMP metabolism was observed in PAO1(∆*rtcB*) as compared to PAO1. In addition, hybrid proteins (harboring both GGDEF and EAL domains) and GGDEF domain-containing proteins were upregulated in PAO1(∆*rtcB*). In contrast, a lower expression of several GGDEF-containing genes was reported in PAO1(∆*rtcB*∆*retS*) than in the PAO1(∆*retS*) strain. To further confirm the data and elucidate the role of c-di-GMP in phenotypical and pathogenic changes of the bacterium after the deletion of *rtcB*, the intracellular content of c-di-GMP was quantified by lysing cells and subsequent analysis of the supernatants using ELISA (Figure 8B). The concentration of c-di-GMP was significantly higher in PAO1(∆*rtcB*) than in PAO1 cultures, whereas no significant difference could be observed in the c-di-GMP content of the PAO1(∆*rtcB*∆*retS*) and PAO1(∆*retS*) strains. Higher concentrations of c-di-GMP in PAO1(∆*rtcB*) as a result of *rtcB* deletion likely account for the increased biofilm formation (Figure 1B) and reporter activity of T6SS (Figure 2B).

## 3. Discussion

In polymicrobial environments, *P. aeruginosa* competes against other colonizing bacteria for its survival [47,48,49]. Various regulatory elements and mechanisms, including quorum sensing, sRNAs, and two-component systems [50], control the pathogenicity and fitness of the bacterium. For the first time, this study introduces the stress response regulator RtcB, a highly conserved protein and part of the bacterial RNA repair operon that seals damaged RNA, as a modulator of *P. aeruginosa* pathogenicity.

To identify the impact of RtcB on the cellular transcriptome of *P. aeruginosa*, RNA-sequencing was performed and compared between PAO1 and PAO1(∆*retS*) backgrounds with and without the deletion of *rtcB*. The obtained results showed that RtcB was involved in modulating global and metabolic cellular pathways, as well as the metabolism of carbohydrates, amino acids, lipids, cofactors, and vitamins. Transmembrane proteins were part of the transcripts increased by *rtcB* deletion and included members of secretion systems. In addition, several virulence factors beyond secretion system-partners were affected by the deletion of *rtcB*, including but not limited to functions involved in pyocyanin production, the secondary messenger c-di-GMP metabolism, biofilm formation, motility, and modulation of quorum sensing pathways and several proteases.

The five secretion systems, T5SS, T2SS, Sec-SRP, T3SS, and T6SS, showed altered expression profiles based on RNAseq data. In *P. aeruginosa*, T2SS and T3SS excrete the greatest number of toxins. Exotoxin A, LasA and LasB proteases, phospholipases H and C, type IV proteases, and lipolytic enzymes are secreted by T2SS [51] and serve in favor of bacterial pathogenicity [52]. Among these effectors, secreted LasB and phospholipase C [53] cause the most harm during lung infections [54]. These exocellular proteins are translocated through the Sec or Tat system’s inner membrane and are subsequently secreted by T2SS or T5SS [55]. T2SS is encoded by two operons, *xcp* and *hxc* [56], and an orphan *xqhA,* which is exclusively expressed when *xcpQ* is mutated [57]. The Sec pathway functions via SecB and Sec-SRP modules [58] and secretes proteins in their unfolded state through the inner membrane with the aid of the SRP particle and FtsY docking protein. T3SS is a hallmark of acute-phase infections by *P. aeruginosa* and excretes ExoSTUY to the eukaryotic host [59]. T5SS is formed from small autotransporters spanned over the cell’s outer membrane [60], organized in six subclasses (Va to Vf) [61]. In PAO1(∆*rtcB*)*,* the expression levels of *paAP* and *cdrA* (members of T2SS and T5SS, respectively) were increased. CdrA is released to the biofilm matrix and promotes biofilm adherence and aggregation even in the absence of Psl exopolysaccharide [62]. In PAO1(∆*rtcB*∆*retS*)*,* T2SS proteins (except for lasB, ToxA, and LipA) were decreased. In addition, the probable Sec-SRP pathway protein PA3822 was increased, while T5SS-related proteins were downregulated in the double mutant.

The exciting finding was the impact of *rtcB* deletion on T3SS and T6SSs. The results showed that RtcB controls the expression of T6SSs (H1-, H2-, H3-T6SS) and T3SS in an opposite manner. Thus, the deletion of *rtcB* confirmed that RtcB suppresses T6SS in PAO1 and T3SS in PAO1(∆*retS*). These data highlight the role of RtcB as a switch controller for the activity of T3SS and T6SSs. To validate the RNA-sequencing data, we selected and quantified various essential genes related to H1-T6SS and T3SS (*rsmA*, *clpV1*, *hcp1*, *exsA*, and *exsC*). *ClpV1* is the cytoplasmic AAA+ ATPase of the H1-T6SS and is necessary for toxin delivery and disassembly of T6SS [39,63]. In *Burkholderia thailandensis*, localization of ClpV-5 in dynamic and discrete foci suggested functional roles beyond solely being an energy provider for T6SS [64]. ExsA is the global regulator of T3SS, binds to the promoters of T3SS-related genes, and upregulates their expression [65]. The expression of *exsC* promotes its antagonistic activity against ExsD and the release of ExsA, resulting in higher expression of T3SS genes [66]. Our RT-PCR quantification results were in line with and validated by RNA sequencing data. Moreover, exploring T3SS genes in PAO1(∆*rtcB*) of RNAseq data revealed that PcrR seems to negatively correlate with this secretory system, which needs to be confirmed in future research.

We examined the involvement of RsmA as a potential mechanism underlying the RtcB-mediated control of T3SS and T6SSs. Immunoblotting indicated that the cellular free RsmA protein was increased in PAO1(∆*rtcB*) and PAO1(∆*rtcB*∆*retS*) as compared to their respective background strains. As the transcription of RsmA showed no change, as evidenced by our RNA-sequencing and RT-PCR data, we envisioned that perhaps the sequestration of RsmA by sRNAs RsmY/Z was reduced, and led to higher RsmA content. Although RNA sequencing showed that transcription of RsmY/Z was not different between strains, deletion of *rtcB* as an RNA processing protein may affect the stability and/or functionality of sRNAs. We bear in mind that the physiological role of RtcB in bacteria remains to be further investigated as there are no tRNA splicing events in bacteria [18,20]. The overexpression of RsmY and RsmZ in the PAO1, PAO1(∆*rtcB*), PAO1(∆*retS*), and PAO1(∆*rtcB*∆*retS*) corroborated that the presence of higher RsmA level is not a consequence of initial lower expression of RsmY/Z, as overexpression of RsmY/Z should theoretically lead to sequestration of RsmA and, consequently, a higher expression of H1-T6SS [46]. In contrast, and to our surprise, the overexpression of RsmY/Z lowered H1-T6SS expression in PAO1(∆*rtcB*). This strongly suggests that RtcB controls the expression of H1-T6SS in PAO1(∆*rtcB*) through a different regulatory pathway that does not directly involve RsmY/Z and RsmA. Our findings did confirm that higher levels of free RsmA protein accounted for the increase of T3SS in PAO1(∆*rtcB*∆*retS*). Finally, active T3SS and T6SS importantly contribute to increased virulence of the RtcB deficient *P. aeruginosa* strains, as evidenced by prokaryotic and eukaryotic competition assays.

Our in vitro findings identify RtcB as a pivotal factor in the pathogenicity and survival of *P. aeruginosa*. Considering that the expression patterns of genes are often different under in vitro and in vivo conditions, our future work will focus on validating the role of RtcB in the pathogenicity of the bacterium by in vivo infection assays using *P. aeruginosa rtcB* deficient strains.

We observed a significant role of RtcB in the modulation of QS genes (Supplementary Materials). The *xcp* of T2SS, and ExsA of T3SS and T6SSs, are regulated by QS-related genes. Generally, the expressions of Las, RhlI, and PQS proteins were increased in PAO1(∆*rtcB*). LasI and RhlI showed higher expression in PAO1(∆*rtcB*∆*retS*), in which the PQS system was repressed. Also, the transcription of *phz* operons was inversely regulated in PAO1(∆*rtcB*) and PAO1(∆*rtcB*∆*retS*) compared to their background strains. Pyocyanin, as a phenotypic marker of the QS system and activity of *phz* operons, was quantified [67]. The production of pyocyanin was manipulated by deletion of *rtcB* and considerably increased in PAO1(∆*rtcB*). Multiple factors, such as higher expression of QS, T2SS, or *phz* operons, could contribute to this observation. In addition, it should be noted that the role of increased transcripts of *phzA2*, *phzB2*, and *phzC2* cannot be neglected in the elevation of pyocyanin production in PAO1(∆*rtcB*).

The cellular c-di-GMP profile was significantly altered by the deletion of *rtcB*. Thus, the metabolism of c-di-GMP was increased in PAO1(∆*rtcB*). A higher expression of GGDEF domain-containing protein was attributed to an overall higher intracellular concentration of c-di-GMP. For instance, *siaD* showed an increase of 2.6-fold in PAO1(∆*rtcB*) compared to PAO1. SiaD contains a GGDEF domain which contributes to synthesizing c-di-GMPs. This was confirmed by a previous study demonstrating that *siaD* deletion lowered intracellular c-di-GMP content [68]. Contrary to PAO1(∆*rtcB*), a lower expression of several GGDEF-containing genes was observed in PAO1(∆*rtcB*∆*retS*) compared to PAO1(∆*retS*). The secondary messenger c-di-GMP plays a crucial role in multiple events that drive virulence, including motility and biofilm formation, and, importantly, the transitions between the sessile and motile bacterial lifestyle. There is no doubt that the considerable elevation in the metabolism of c-di-GMP can be contributed to the notable shift in genotypic and phenotypic features in PAO1(∆*rtcB*) and PAO1(∆*rtcB*∆*retS*).

## 4. Materials and Methods

### 4.1. Bacterial Strains, Growth Conditions, and Antibiotic Reagents

Bacterial strains and plasmids used in this study are presented in Table 2. *Escherichia coli* and *Pseudomonas aeruginosa* were routinely grown at 37 °C on LB agar or in LB broth unless indicated otherwise. The concentration of antibiotics used are as follows: for *E. coli* cultures: gentamycin (Gm) at 15 µg mL^−1^, tetracycline (Tc) at 12.5 µg mL^−1^, kanamycin (Kn) at 50 µg mL^−1^, and ampicillin (Ap) at 50 µg mL^−1^; for *P. aeruginosa* cultures: Gm at 150 µg mL^−1^ in Pseudomonas isolation agar (PIA) and 50 µg mL^−1^ in LB, carbenicillin (Cb) at 250 µg mL^−1^ in LB, Tc at 200 µg mL^−1^ in PIA and 70 µg mL^−1^ in LB, and trimethoprim (Tmp) at 300 µg mL^−1^ in LB.

### 4.2. Construction of Gene Expression Reporters

The promoterless plasmid pMS402 carrying the *luxCDABE* gene cluster was used for the construction of reporter fusion of desired genes [70]. Primers used in this study are presented in Table 3. The promoter of H1-T6SS operon and *exoS* was PCR-amplified and cloned into the *Bam*HI-*Xho*I site upstream of the *lux*-Box on pMS402 plasmid, resulting in the so-called pKDH1 reporter. The *Pac*I digested fragment of pKDH1 was cloned into integration plasmid CTX6.1, originated from mini-CTX-*lux* to construct a chromosomally integrated reporter [74]. CTXH1 was transferred to *E. coli* SM10-λ *pir* [69]*,* and biparental-mating between *E. coli* SM10-λ *pir, Pseudomonas* recipients was set up to construct the H1-T6SS reporter strain [75,76]. For the construction of other reporters, the same strategy was applied. Gene expression measurements were carried out by quantifying the light production (luminescence per second-LPS) of the reporter strain using Synergy2 Multimode Microplate Reader (BioTek). Bacterial growth was recorded at OD_600_ during the luminescence readout. The measurement continued for 24 h with 30 min intervals at 37 °C. Reporter activity graphs are shown at the 12 h time point when bacteria enter the stationary phase of growth and reporter activity remains stable.

### 4.3. Construction of Mutant Strains

The *sacB*-based method was applied to construct the knockout mutants, as previously described [72]. In brief, approximately 1 kb of each upstream and downstream region of the desired gene was PCR-amplified with the incorporation of restriction sites. The primers used in this study are listed in Table 3. The PCR products were cloned into suicide vector pEX18Tc. Triparental mating was set up using *E. coli* strains carrying helper plasmid pRK2013, pEX18Tc carrier, and PAO1 and PAO1(*ΔretS*) to generate mutant strains [71]. The mutants were confirmed by PCR and agarose gel electrophoresis.

### 4.4. Pyocyanin Assay

Bacteria were grown overnight for 16 h after which supernatants were collected. The pyocyanin content was measured according to a previously described protocol [77]. Five mL of supernatant was mixed with 3 mL chloroform. The chloroform layer was separated by centrifugation and transferred into a fresh falcon tube. One mL of 0.2 N HCL was added to the falcon tubes and mixed well, following centrifugation at 5488× *g* for 10 min. The top layer was separated, and its absorbance was measured at 520 nm. The cellular content of pyocyanin was presented as µg pyocyanin per mL supernatant, calculated by multiplying the extinction coefficient of 17.072 at 520 nm.

### 4.5. Biofilm Formation and Bacterial Motility Assay

Static biofilm formation was assessed as previously described by O’Toole and Kolter [78]. A dilution ratio of 1:100 was made from an overnight culture (37 °C) of bacteria into 96-well polystyrene microtiter plates (Costar), which were subsequently incubated for 24 h at 37 °C. The cultures were rinsed with phosphate-buffered saline (PBS) three times and stained with 1% crystal violet for 20 min at room temperature. After rinsing with distilled water, 150 µL 2 mM acetic acid was added to each well to dissolve the remaining crystal violet; 100 µL of this solution was transferred to a new microtiter plate and the absorbance was read at 550 nm (OD_550_).

Motility was assessed as described previously [79]. Three different media compositions were made for swarming (0.5% agar, 5 gl^−1^ glucose, 8 gl^−1^ nutrient broth mix), swimming (0.3% agar, 5 gl^−1^ NaCl, 10 gl^−1^ tryptone), and twitching (LB broth with 1% agar). Overnight cultures (2 µL) of bacteria were spotted on the surface of swarming and swimming plates and stabbed into twitching plates. Swarm plates were incubated at 37 °C, swim plates at 30 °C for 15 h, and twitch plates at 37 °C for 24 h. Images of all plates were obtained using the Fusion FX imaging system.

### 4.6. RNA Isolation and RNA-Sequencing and Analysis of Differentially Expressed Genes

Strains were grown overnight at 37 °C for 16 h followed by sub-culturing into fresh LB medium for 6 h (to mid-exponential phase). RNAlater stabilization solution (invitrogen^TM^) was added to the samples to fix the RNAs. Total RNA was extracted from PAO1, PAO1(*ΔrtcB*), PAO1(*ΔretS*), and PAO1(*ΔrtcBΔretS*) by TRIzol-based method (Life Technologies, Carlsbad, CA, USA). Briefly, the sub-cultures were centrifuged at 12,000× *g* for 5 min. Pellets were resuspended in 1 mL of TRIzol and incubated at room temperature for 5 min. Then, 0.2 mL of chloroform were added into the tubes and vortexed, following centrifugation. The upper aqueous phase from each strain was transferred to a fresh tube and 0.5 mL of cold isopropanol was added to precipitate RNA. After centrifugation (12,000× *g* for 10 min), the pellets were resuspended in 1 mL 75% ethanol and centrifuged. The RNA pellets were air-dried and dissolved in 50 µL RNase-free water.

RNA-sequencing analysis was carried out by GENE DENOVO, China. Consecutively, rRNA was depleted from 1 µg of the total RNA using the Ribo-Zero Magnetic Gold Kit (Epicentre Biotechnologies, Madison, WI, USA). The complementary DNC (cDNA) library was constructed, and samples were sequenced using the Illumina Hiseq^TM^ 2500 platform with paired-end 150 base reads. The raw data were filtered and reproducibility between samples was evaluated. The gene expression level was normalized using the fragments per kilobase of transcript per million mapped reads (FPKM) method. Differentially expressed genes (DEGs) in samples with a fold change ≥ 2 and q-value < 0.05 were identified using the edgeR package (http://www.r-project.org/; accessed on 19 January 2019). Ultimately, DEGs were used for enrichment analysis of KEGG and GO pathways. Experiments were carried out using three independent biological replicates for each strain.

### 4.7. Quantitative Real-Time PCR Analysis

Single colonies of each desired strain were grown (three times) overnight in 2 mL LB broth at 37 °C. Following 3 h subculture in 2 mL fresh LB broth, 1 mL aliquots of the cell cultures were harvested at the mid-exponential phase. RNeasy bacteria protect solution (Qiagen, Hilden, Germany) was added to each aliquot and total RNA was isolated using TriRNA Pure Kit (Geneaid, New Taipei, Taiwan) according to the manufacturer’s protocol. Eluted total RNA was purified a second time by RNA Cleanup Kit (Geneaid) with a residual DNA elimination treatment step. DNase was deactivated by the addition of ethylene diamine tetra-acetic acid (EDTA). Following the manufacturer’s instructions, cDNA was synthesized using Maxima First Strand can Synthesis Kit for RT-qPCR with dsDNase (Thermo Fisher Scientific, Waltham, MA, USA). Also, a set of reverse transcriptase minus (RT-) negative controls was amplified to confirm the lack of gDNA contamination. The mRNA levels were measured using the PowerUp SYBR Green Master Mix (Thermo Fisher Scientific) and an Illumina real-time PCR system by following the manufacturer’s instructions. For data interpretation, mRNA levels of the desired genes were normalized to the expression of the *rpoD* housekeeping gene, and fold change was calculated using the 2^−∆∆Ct^ method [80]. Primers used for qRT-PCR analysis are listed in Table 2.

### 4.8. Bacterial Competition Assay

To evaluate activated T6SSs (H1-, H2-T6SS) in PAO1 strains, an in vitro bacterial competition assay was carried out as described previously with minor modifications of the protocol [81]. *E. coli* DH5α expressing β-galactosidase was chosen as the prokaryote prey. PAO1, PAO1(*ΔrtcB*), PAO1(*ΔretS*), and PAO1(*ΔrtcB*Δ*retS*) strains were co-incubated with prey as spots on filter papers on LB agar at 37 °C. After a 5 h incubation, each filter paper was resuspended in cold PBS and washed three times. Samples were serially diluted from 10 to 10^−5^ and 5 μL of each dilution was spotted on a coliform agar plate for the visual assay. After incubating the plates at 37 °C for 24 h, LacZ-positive *E. coli* abundance was assessed by counting the blue-colored colonies. A fluorometric competition assay was performed with *E. coli* harboring the *pilG*-DsRed expressing vector, emitting red fluorescence to confirm the competition. Slides were prepared from post-co-incubation of prey and predator, and inspected using an inverted microscope (Nikon ECLIPSE T*i*-E).

### 4.9. Plant Virulence Assay

The plant infection assay was performed in lettuce leaves as described previously [82]. Bacterial cultures were grown overnight, adjusted to OD_600_ = 0.01, and washed three times with cold PBS buffer. The pellets were resuspended in 10 mM MgSO_4_ and used to inoculate the mid-ribs of romaine lettuce leaves. The inoculated leaves were incubated at room temperature for 2 days in plates containing a Whatman filter paper soaked in 10 mM MgSO_4_. The leaves were closely monitored during the incubation and imaged at 48 h.

### 4.10. Immunoblotting Assay

*P. aeruginosa* strains were grown in 2 mL LB broth at 37 °C for 12h. Bacteria were spun down by centrifugation and the pellet was washed 3 times with cold 1X PBS. A 20 µL protease inhibitor cocktail (Abcam, Cambridge, UK) and 1 mL of 0.6 M perchloric acid were added to the pellet, after which cells were resuspended and stored on a rotatory shaker for 30 min at 4 °C. Following centrifugation at 16,128× *g* for 10 min at 4 °C, pellets were resuspended in 6 M urea and protein concentrations were determined by the Bradford assay [83]. Equal amounts of protein from the prepared samples were subjected to SDS-PAGE at 100 V on 15% polyacrylamide gels for approximately 1h. Polyvinylidene difluoride (PVDF) membranes were activated in methanol and placed in a transfer buffer. Next, proteins were transferred to PVDF membranes, which were subsequently blocked for 90 min using a TBST buffer containing 5% non-fat milk. Membranes were then washed with TBST buffer and probed overnight (4 °C) using 1:500 dilution (TBST+5% non-fat milk) of CsrA- *E. coli* antibody (rabbit). After incubation, membranes were washed 3 times for 20 min with TBST buffer and subsequently incubated with anti-rabbit IgG antibody (1:1000 dilution in TBST+1% non-fat milk) for 1 h followed by 3 washes (20 min each) on a rotatory shaker. Protein bands were visualized using an enhanced chemiluminescence (ECL) kit. To confirm equal loading for all samples, membranes were stained with Ponceau S for 10 min. The Fusion FX imaging system was used to obtain images.

### 4.11. Construction of Overexpression Vectors

The multi-copy-number *E. coli*-*P. aeruginosa* shuttle vector pAK1900 carrying a *lac* promoter was used to express the RtcB in trans [84]. The entire DNA region of these genes was PCR-amplified in-frame, with the incorporation of restriction sites. The primers used are listed in Table 2. PCR products were cloned into *Bam*HI and *Hind*III sites of pAK1900 and transferred into *Pseudomonas* by electroporation.

### 4.12. C-di-GMP Quantification

C-di-GMP was quantified using a CdiGMP Elisa Kit from Mybiosource (#MBS288159). To determine intracellular c-di-GMP content in cultures, we extracted c-di-GMP from bacterial cells following a previously described protocol with some modifications [85]. In brief, bacterial strains were grown in 5 mL LB broth for 24 h at 37 °C and bacterial density was adjusted to OD_600_ 1.8. Cells were pelleted down for 7 min at 1792× *g* and washed three times with PBS buffer. Next, 250 µL of 0.6 M perchloric acid was added to the washed pellet, followed by a 30 min incubation at 4 °C on a rotatory shaker to ensure proper lysis. Following centrifugation at 18,928× *g* for 10 min, the supernatant was transferred to a fresh Eppendorf tube. Two hundred µL of 1M KOH was added to the tubes, and the salt by-product was settled by centrifuging at 18,928× *g* for 10 min. The clear supernatant was used for the quantification of c-di-GMP according to the kit manual.

### 4.13. Statistical Analysis

Statistical analysis was performed using unpaired student’s *t*-test or one-way analysis of variance (ANOVA) with Dunnett post hoc tests. All data represent means ± SEM from independent experiments. Where applicable, the statistical significance is: **** p < 0.0001; *** p < 0.001; ** p < 0.01; * p < 0.05; NS *p* > 0.05.

## Figures and Tables

**Table 1:** Differential expression of secretory proteins of T2SS and T5SS.

Gene Locus	Secretion System	Secreted Protein	*p* Value	FDR	Log 2-Fold Change
	PAO1(∆*rtcB*)				
PA2939	T2SS	PaAP	6.97 × 10^−15^	3.55 × 10^−13^	1
PA4625	T5SS	CdrA	5.24 × 10^−43^	1.53 × 10^−40^	2.1
	PAO1(∆*rtcB*∆*retS*)				
PA0572	T2SS	Protease	2.25 × 10^−15^	4.64 × 10^−14^	−1.5
PA0852	T2SS	CbpD	4.32 × 10^−21^	1.18 × 10^−19^	−1.6
PA1249	T2SS	AprA	5.56 × 10^−6^	4.76 × 10^−5^	−1.2
PA1948	T2SS	ToxA	5.76 × 10^−6^	4.91 × 10^−5^	1.1
PA2862	T2SS	LipA	1.08 × 10^−7^	1.17 × 10^−6^	4.8
PA2939	T2SS	PaAP	3.42 × 10^−17^	7.92 × 10^−16^	−1.5
PA2676	T2SS	HplS	0.010726	0.041385	−1.8
PA2677	T2SS	HplR	5.62 × 10^−8^	6.40 × 10^−7^	−3
PA2678	T2SS	Unknown protein	1.53 × 10^−5^	0.000119	−3.3
PA3105	T2SS	XcpQ	1.43 × 10^−23^	4.40 × 10^−22^	−2.1
PA3724	T2SS	LasB	1.48 × 10^−11^	2.35 × 10^−10^	1.2
PA3822	Sec-SRP	Unknown protein	1.14 × 10^−5^	9.19 × 10^−5^	1
PA4082	T5SS	CupB5	2.84 × 10^−11^	4.39 × 10^−10^	−2.3
PA4624	T5SS	CdrB	1.47 × 10^−8^	1.82 × 10^−7^	−1.3
PA5210	T2SS	Probable ATPase	3.32 × 10^−9^	4.30 × 10^−8^	−1.1

**Table 2:** Bacterial strains and plasmids used in this study.

Bacterial Strains or Plasmid	Relevant Characteristics/Sequence	Source
*E. coli* strains		
DH5α	F^−^ φ80*lac*Z∆M15 ∆(*lacZYA*-*argF*) U169 *recA*1 *endA*1 *hsdR*17 (r_k_^−^, m_k_^+^) phoA supE44 *λ*^−^ *thi*^−^1 *gyrA*96 *relA*1	Invitrogen
SM10-λ *pir*	Mobilizing strain, RP4 integrated into the chromosome; Kn^r^	[69]
Mach1™-T1^R^	F- Φ80*lac*ZΔM15 Δ*lac*Χ74 *hsd*R(rK- mK+) Δ*rec*A1398 *end*A1 *ton*A	Invitrogen
*P. aeruginosa* strains		
PAO1	Wild type, lab strain	In-house
PAO1 (∆*retS*)	*retS* replacement mutant of PAO1	In-house
PAO1 (∆*rtcB*)	*rtcB* replacement mutant of PAO1	This study
PAO1 (∆*rtcB*∆*retS*)	*rtcB retS* replacement mutant of PAO1	This study
Plasmids		
pMS402	Expression reporter plasmid carrying the promoterless *luxCDABE*; Kn^r^ Tmp^r^	[70]
CTX-6.1	Integration plasmid origins of plasmid mini-CTX-*lux*; Tc^r^	In-house
pRK2013	Broad-host-range helper vector; Tra^+^, Kn^r^	[71]
pEX18Tc	*oriT*^+^*sacB*^+^ gene replacement vector with multiple-cloning site from pUC18; Tc^r^	[72]
pAK1900	*E. coli*-*P. aeruginosa* shuttle cloning vector, Amp^r^	[73]
pAK-*rtcB*	pAK1900 with a 1260 bp fragment of PA4583 between *Bam*HI and *Hin*dIII; Amp^r^, Cb^r^	This study
pEX18Tc-*rtcB*	pEX18Tc carrying the upstream and downstream fragment of *rtcB*	This study
pKD-H1T6SS	pMS402 containing H1-T6SS promoter region; Kn^r^, Tmp^r^	In-house
CTX-H1T6SS	Integration plasmid, CTX6.1 with a fragment of pKD-H1-T6SS containing H1 promoter region and *luxCDABE* gene; Kn^r^, Tmp^r^, Tc^r^	This study
CTX-ExoS	Integration plasmid, CTX6.1 with a fragment of pKD-ExoS containing *exoS* promoter region and *luxCDABE* gene; Kn^r^, Tmp^r^, Tc^r^	In-house
pAK-*rsmY*	pAK1900 with a 124 bp fragment of *rsmY* between *Bam*HI and *Hin*dIII; Amp^r^, Cb^r^	This study
pAK-*rsmZ*	pAK1900 with a 116 bp fragment of *rsmZ* between *Bam*HI and *Hin*dIII; Amp^r^, Cb^r^	This study

**Table 3:** Primers used in this study.

Primer	Sequence (5′→3′) ^a^	Restriction Site
*rtcB* -UP-S	acagcgGAATTCCTGGTGGAGCGCTACTTCACC	*Eco*RI
*rtcB* -UP-AS	acagcgGGATCCGTCCAGAGCTTGATCGGCTT	*Bam*HI
*rtcB* -DW-S	acagcgGGATCCCGATGGCCTACAAGGACATCG	*Bam*HI
*rtcB* -DW-AS	acagcgAAGCTTGATGTTGCGGATGCGCAGC	*Hin*dIII
pAK-*rtcB*-S	acagcgAAGCTTGGAGCACAAGGAAAGCACGATG	*Hin*dIII
pAK-*rtcB*-AS	acagcgGGATCCCGTCATCCTTTCACGCACACC	*Bam*HI
pKD-*rtcB*-S	acagcgCTCGAGGCTCGAACGTTGCCTTAACGGC	*Xho*I
pKD-*rtcB*-AS	acagcgGGATCCGGAATACAGCAGGCCGCGTTCG	*Bam*HI
pAK-*rsmY*-S	acagcgAAGCTTCATGCTGGGAAGGCTCGCGATG	*Xho*I
pAK-*rsmY*-AS	acagcgGAATTCCTGAAGGTCGGCCTGGTCTACC	*Bam*HI
pAK-*rsmZ*-S	acagcgGGATCCGTGACGCGCTGTTCCAGTGACG	*Xho*I
pAK-*rsmZ*-AS	acagcgGGAATTCATCGAGCTGAACAGC	*Bam*HI
*rpoD*-S	GATCTCCATGGAAACCCCGATC	
*rpoD*-AS	GAGGACTTCGCGGGTGGATTC	
*exsA*-S	TTCTGCTCGAGGGCGAACTGAC	
*exsA*-AS	CGGCTGTCCTTTCCCTTGGTAC	
*clpv1*-S	CTGAACAGCCTGGCCTACAAGG	
*clpv1*-AS	GAGTCCGGCAACTGGAGGATC	
*hcp1*-S	GACGTCAAGGGTGAGTCCAAGG	
*hcp1*-AS	CAGGTTGGGCGTGGACTTGTC	
*exsC*-S	CAAGGTCAACCGACTGCTTGC	
*exsC*-AS	CATCGGCCTCCAGCAACAGAC	
*rsmA*-S	AGACCCTGATGGTAGGTGACGA	
*rsmA*-AS	TGGATGCGCTGGTAAATTTCCT	

^a^, underlined are restriction site sequences. The overhanging base pairs are shown with lower case.

## Data Availability

Not applicable.

## Supplementary Materials

The following are available online at https://www.mdpi.com/article/10.3390/ijms222212561/s1.
